# Autistic adults’ experiences of financial wellbeing: Part II

**DOI:** 10.1177/13623613231191594

**Published:** 2023-10-05

**Authors:** Elizabeth Pellicano, Gabrielle Hall, Ru Ying Cai

**Affiliations:** 1 University College London, UK; 2 Macquarie University, Australia; 3 Cooperative Research Centre for Living with Autism (Autism CRC), Australia; 4 Aspect Australia, Australia

**Keywords:** employment, income, mental health, money, quality of life

## Abstract

Financial wellbeing is an important component of people’s overall wellbeing, reflecting the capacity to live a comfortable and fulfilling life. Yet virtually nothing is known about this topic for autistic people. This study addressed this gap using a two-phase sequential mixed-methods design. Here, we report on findings from the Phase 2 qualitative study. In this study, 21 autistic participants were purposively selected based on the status of their financial wellbeing, including 12 people with high, and 9 with low, financial wellbeing, and interviewed by an autistic researcher. We identified four themes through reflective thematic analysis. Having access to a stable income made an enormous difference to people’s financial wellbeing. Participants emphasised how their broader social supports, especially family support, shaped their financial wellbeing. Yet, planning financially was often challenging, especially for people who had insufficient money to meet their basic needs. Nevertheless, our participants reported a strong drive to stay in control and avoid unnecessary risk. Our findings revealed how much money matters in autistic people’s lives. Future research should investigate the ways in which autistic people could secure more reliable incomes and identify the most effective ways to support them to achieve financial resilience.

**Lay abstract:**

Money matters in people’s lives. It helps to meet people’s basic needs (food, clothes, shelter) and live the lives they want to. When people talk about ‘financial wellbeing’, they mean how much you feel in control over day-to-day finances and how much freedom you have to make choices to enjoy life. We don’t know what autistic people think about these things. That’s why we did our study. We spoke to 21 autistic adults (24–69 years) about how they felt about their financial situation. We deliberately spoke to people who had told us previously they felt ‘financially well’ or ‘financially unwell’ so we could hear a range of opinions. Autistic people told us financial wellbeing meant having enough money to pay for their basics needs, to have a safety net for unexpected bills and not having to worry about money now or in the future. But many felt that good financial wellbeing was not possible for them. They often did not have a stable income to cover day-to-day expenses. This limited the choices they could make. Despite these challenges, autistic people told us they worked hard to budget and save money when they could – because feeling financial insecure was just too stressful, especially when they could not rely on family or friends for support. It made them feel mentally unwell. Our study shows there are many factors that influence autistic people’s financial wellbeing. We need more research to help us understand how autistic people can be supported to achieve financial security.

Money plays a crucial role in most people’s quality of life (Netemeyer et al., 2018). In the context of economic instability and rising costs, the concept of ‘financial wellbeing’ has received growing attention (Taylor et al., 2011). While there is much debate about what financial wellbeing means (e.g. Brüggen et al., 2017; Salignac et al., 2019), there is general consensus that it is more than just about how much money a person has; it is also about the ways in which people use that money now and in the future (Consumer Financial Protection Bureau (CFPB), 2019; Kempson et al., 2017; Mahendru et al., 2022; Netemeyer et al., 2018). Here, we focus on a group of people whose financial wellbeing might be particularly disadvantaged, autistic adults. We sought to examine their subjective experiences of financial wellbeing and identify which factors shape these experiences.

## Financial wellbeing in the general population

Conceptualisations of financial wellbeing are intimately tied to notions of wellbeing per se, the subjective assessment of how one perceives themselves to be getting on in their life (Deci & Ryan, 2008). Although early research focused on objective measures (e.g. income, or debt-to-income ratio) as indicators of a person’s – or a population’s – financial wellbeing (Browning & Lusardi, 1996; Easterlin, 1995; Ng & Diener, 2014; Schmeiser & Seligman, 2013; Soman & Cheema, 2002), researchers have since shifted to subjective measures (e.g. of peoples’ perceptions about and responses to their financial circumstances), or a combination of the two. This shift acknowledges that people in objectively-similar financial situations might perceive their financial wellbeing differently (Brüggen et al., 2017). People on low incomes, for example, do not always report financial strain (Hazelrigg & Hardy, 1997; Mirowsky & Ross, 1999).

Most recent approaches to financial wellbeing highlight a sense of control (Bowman et al., 2017) and suggest it is comprised of three main components: being able to meet financial obligations, having enough money left over to enjoy life and having the financial resilience to cope with unexpected expenses (Kempson et al., 2017; see also CFPB, 2019). Consistent with this conceptualisation, studies have repeatedly demonstrated that a person’s income *and* their financial behaviours (e.g. budgeting, active saving, not borrowing for everyday expenses) are related to subjective financial wellbeing (Australia and New Zealand Banking Group [ANZ], 2018; Joo & Grable, 2004; Kempson et al., 2017; Russell et al., 2020; Shim et al., 2009). Personal characteristics such as gender, age and education (Boshara et al., 2015; Joo & Grable, 2004; Malone et al., 2010) – as well as having a disability that restricts a person’s everyday activities (Comerton-Forde et al., 2018) – also affect one’s assessment of financial wellbeing.

There is also growing recognition that such assessment is not driven solely by individual factors. Instead – and consistent with ecological models (Bronfenbrenner, 1994) – there is a complex interplay between a person’s financial wellbeing and the broader context, including across individual-, household-, community- and societal-levels (Salignac et al., 2020). At the household level, families and friends can play a strong role in shaping an individual’s financial wellbeing, through family/caring responsibilities, couple dynamics, parents’ financial attitudes and behaviours and comparisons with peers. These elements, together with individual factors, are in turn shaped by broader community- and societal-level factors, including cost of living, government policy, or access to financial products and services. Understanding an individual’s sense of financial wellbeing within this context is critical to addressing poor financial wellbeing – not least because it has been linked to financial hardship (Arber et al., 2014; Muir et al., 2017), poor health (Arber et al., 2014; Benzeval et al., 2014), relationship instability (Arber et al., 2014), mental distress (Haisken-DeNew et al., 2018; see Hassan et al., 2021), and mental and physical health (ANZ, 2021).

## Financial wellbeing in autistic people

All this knowledge comes from research with the general population. We know strikingly little about the financial wellbeing of autistic people. There are at least three reasons to expect that autistic people’s financial wellbeing may be compromised. First, despite many autistic people reporting a desire to work, the majority are un- or underemployed (C. Anderson et al., 2021; Nicholas & Klag, 2020) and consequently have low levels of income (Cai, Gallagher, et al., 2023; Russell et al., 2017). Also, autistic people have a substantially elevated risk of experiencing mental health problems, especially anxiety, depression (Hollocks et al., 2019; Hudson et al., 2019) and physical health issues (Rydzewska et al., 2021). These compounding issues, along with fatigue and burnout (Higgins et al., 2021; Raymaker et al., 2020), may also impact their capacity to obtain and sustain employment. Second, research has demonstrated consistently that, compared to non-autistic people, autistic adults typically fare badly both on conventional quality of life assessments (Ayres et al., 2018) and on standard ‘life achievements’, like living independently or having friends and intimate relationships (Howlin & Magiati, 2017) (see Pellicano, Hall, et al., 2022, for review). Given that financial wellbeing has been linked to overall wellbeing (Netemeyer et al., 2018), it seems plausible that autistic people’s poorer quality of life and wellbeing – at least as rated using standard, researcher-defined measures – should extend to the financial domain. Third, autistic people can have difficulties with executive function, including planning, organising, prioritising (St John et al., 2022), and can depend significantly on support from others – at home, school, work and in the community. These issues may hinder the degree of control necessary to achieve and maintain financial wellbeing.

Our initial research showed that many autistic adults indeed have low levels of financial wellbeing. In Phase 1 of a two-phase mixed-methods study, we surveyed 191 Australian autistic adults about their financial wellbeing and its predictors (Cai, Hall, & Pellicano, 2023). Compared to a general population sample (ANZ, 2018), our participants reported poorer subjective financial wellbeing, with almost half indicating they were ‘struggling’ or just ‘getting by’. We also showed objectively that participants’ annual income was markedly low compared to the average full-time Australian salary, with almost half reporting annual earnings at, or below, the poverty line (see also Cai, Gallagher, et al., 2023; Roux et al., 2015; Zwicker et al., 2017). The predictors of financial wellbeing in our autistic sample were consistent with those in the general population (ANZ, 2018; Comerton-Forde et al., 2018; Muir et al., 2017), with income *and* financial behaviours contributing to autistic adults’ subjective financial wellbeing: the more people were able to save and the less that they had to borrow for everyday expenses, the greater their financial wellbeing. There was one exception, however: depressive symptoms also significantly predicted autistic adults’ subjective financial wellbeing, indicating the important (and likely complex) inter-relationships between mental health and financial wellbeing (Arber et al., 2014).

## The current study

The results from Phase 1 provide important initial insights into autistic adults’ financial wellbeing (Cai, Hall, & Pellicano, 2023). Notably, however, that phase focused solely on individual factors. In Phase 2 of our mixed-methods study, reported herein, we invited a subset of Phase 1 survey participants with self-reported high or low financial wellbeing to participate in an in-depth, qualitative interview. Specifically, we sought to understand their experiences of financial wellbeing, and to identify the individual and broader social factors (cf. Salignac et al., 2020) that might support autistic people to achieve good financial wellbeing.

## Method

### Design

This study followed a two-phase explanatory sequential mixed-methods design (participant selection model) (Ivankova et al., 2006). In Phase 1, autistic adults recruited via convenience sampling, including through community contacts and social media, were invited to participate in an online, quantitative survey (Cai, Hall, & Pellicano, 2023). Participants needed to have a formal diagnosis of autism or self-identify as autistic (to account for the often-prohibitive costs and/or lengthy delays in gaining a clinical diagnosis; Howlin, 2021), be aged ⩾ 18 years and live in Australia. The survey included a measure of participants’ subjective financial wellbeing (Kempson et al., 2017), which asked about (1) people’s ability to meet financial commitments; (2) the extent to which they feel comfortable about their current and future financial situation; and (3) their financial resilience. Participants received scores out of 100 for each, which were averaged to yield a total subjective financial wellbeing score (ranging from 0 to 100) (Cai, Hall, & Pellicano, 2023). Following the Phase 1 survey, participants were invited to participate in Phase 2, a semi-structured interview. Of those who expressed interest, we purposively selected participants who had obtained subjective financial wellbeing scores in the lower (0–25) or upper (75–100) quartiles, thus ensuring that we elicited a diversity of views.^1^

### Participants

One-hundred-and-ninety-one autistic participants took part in Phase 1 (Cai, Hall, & Pellicano, 2023). Thirty-two participants expressed interested in being involved in Phase 2, including 15 with high and 17 with low subjective financial wellbeing.

#### High financial wellbeing group

Of the 15 people with high financial wellbeing scores (hereafter, ‘high group’) who had expressed interest, 12 (80%) completed an interview. They ranged from 28 to 69 years and had received an independent clinical diagnosis of an autism spectrum condition, on average, at 36 years (Table 1). Only two participants had received their autism diagnosis during childhood; one of these participants had also received an intellectual disability diagnosis and was supported by their carer during the interview. None reported preferring or predominantly using non-traditional forms of communication. Most participants were university educated (*n* = 8; 67%), identified as women (*n* = 9; 75%) and were of white ethnic background (*n* = 11; 92%). Five (42%) reported having one or more children, with at least one autistic child (Table 1).

**Table 1.** Participant characteristics for the low and high financial wellbeing groups.

	Low financial wellbeing group (*n* = 9)	High financial wellbeing group (*n* = 12)	Total (*n* = 21)
	Mean (*SD*) Range or *N* (%)		
Age (in years)	36.67 (13.56) 24–56	45.00 (11.18) 28–69	41.43 (12.66) 24–69
Age of autism diagnosis (in years)	29.56 (13.43) 3–52	36.33 (15.13) 3–52	33.43 (14.48) 3–52
AQ-Short^a^	87.22 (10.76) 73–110	81.92 (12.92) 63–99	84.19 (12.05) 63–110
Gender			
Woman	5 (55%)	9 (75%)	14 (67%)
Man	4 (44%)	3 (25%)	7 (33%)
Ethnic background			
White European	7 (77%)	11 (92%)	18 (86%)
White Other	2 (22%)	0	2 (10%)
South-East Asian	0	1 (8%)	1 (5%)
Living arrangements			
Alone	1 (11%)	3 (25%)	4 (19%)
Spouse/partner	2 (22%)	3 (25%)	5 (24%)
Spouse/partner and children	0	3 (25%)	3 (14%)
With parents	2 (22%)	1 (8%)	3 (14%)
With children only	3 (33%)	1 (8%)	4 (19%)
With other relatives	1 (11%)	1 (8%)	2 (10%)
Number of children			
One child	1 (11%)	2 (17%)	3 (14%)
Two children	1 (11%)	2 (17%)	3 (14%)
Three children	1 (11%)	1 (8%)	2 (10%)
Four children	1 (11%)	0	1 (5%)
Number of autistic children			
No autistic children	2 (22%)	0	2 (10%)
One autistic child	1 (11%)	4 (33%)	5 (24%)
Two autistic children	1 (11%)	0	1 (5%)
Three autistic children	0	1 (8%)	1 (5%)
Highest level of education			
Started high school	2 (22%)		2 (10%)
College certificate or diploma	3 (33%)	4 (33%)	7 (33%)
University degree	4 (44%)	8 (67%)	12 (57%)
Employment status			
Studying	1 (11%)	0	1 (5%)
Studying and (self)employed	2 (22%)	2 (17%)	4 (19%)
Employed/self-employed	1 (11%)	9 (75%)	10 (48%)
Not currently employed/studying	5 (55%)	1 (8%)	6 (28%)
NDIS plan^b^	5 (55%)	2 (17%)	7 (33%)
Self-reported co-occurring diagnoses			
ADHD	0	3 (25%)	3 (14%)
Anxiety disorder	4 (44%)	5 (42%)	9 (43%)
Bipolar disorder	2 (22%)	0	2 (10%)
Chronic fatigue	0	1 (8%)	1 (5%)
Chronic pain	1 (11%)	3 (25%)	4 (19%)
Depression	5 (55%)	7 (58%)	12 (57%)
Diabetes	1 (11%)	1 (8%)	2 (10%)
Drug/alcohol dependence	1 (11%)	1 (8%)	2 (10%)
Dyslexia	0	1 (8%)	1 (5%)
Dyspraxia	0	1 (8%)	1 (5%)
Eating disorders	3 (33%)	2 (17%)	5 (24%)
Epilepsy	0	1 (8%)	1 (5%)
Gastrointestinal issues	1 (11%)	4 (33%)	5 (24%)
Hypertension	1 (11%)	2 (17%)	3 (14%)
Intellectual disability	0	1 (8%)	1 (5%)
Obesity	2 (22%)	4 (33%)	6 (28%)
OCD	0	1 (8%)	1 (5%)
Personality disorder	3 (33%)	0	3 (14%)
PTSD	3 (33%)	1 (8%)	4 (19%)
Schizophrenia	0	1 (8%)	1 (5%)
Sleep disorders	4 (44%)	6 (50%)	10 (48%)
Stroke	0	1 (8%)	1 (5%)
PHQ-9 (depression)^c^	19.22 (7.64) 3–27	9.92 (6.29) 0–21	13.91 (8.21) 0–27
DSM-5 GAD-D (anxiety)^c^	24.78 (8.39) 7–34	13.08 (8.16) 0–27	18.09 (10.00) 0–34

AQ: Autism Spectrum Quotient; NDIS: National Disability Insurance Scheme; ADHD: Attention Deficit Hyperactivity Disorder; OCD: Obsessive Compulsive Disorder; PTSD: Post Traumatic Stress Disorder; PHQ-9: Patient Health Questionnaire–9 (Kroenke et al., 2001); DSM-5 GAD-D: DSM-5 Generalised Anxiety Disorder Dimensional Scale (Knappe et al., 2013).

a AQ-Short (Hoekstra et al., 2011), where higher scores reflect greater autistic features; ^b^Australia’s NDIS gives disabled people access to funding for supports and services; ^c^Higher scores on the PHQ-9 and DSM-5 GAD-D are indicative of greater depression and anxiety, respectively.

#### Low financial wellbeing group

Of the 17 people with low financial wellbeing scores (hereafter, ‘low group’) who had expressed interest, nine (53%) completed an interview (the remaining eight did not respond to follow-up emails). Participants ranged in age from 24 to 56 years and seven (78%) had received a formal diagnosis of autism, almost all (*n* = 6; 86%) in adulthood. Two adults self-identified as autistic. Both also obtained scores above the cut-off of 65 on the Autism Spectrum Quotient–Short (AQ-Short; Hoekstra et al., 2011) (Table 1). All participants identified as white, five (55%) as women and four (44%) had obtained a university degree. Four (44%) reported having one or more children; two with autistic children. None of our participants in the low group reported having an intellectual disability or being unable to use traditional forms of communication.

### Materials and procedure

Ethical approval for the study was obtained from the Macquarie University’s Human Research Ethics Committee (Reference number: 52019572512775). All participants provided written, informed consent prior to interview.

#### Quantitative measures

With interviewees’ prior permission, we extracted relevant data from our Phase 1 survey to give broader context to their responses. In the survey, we asked about participants’ demographic characteristics and co-occurring conditions (Table 1), their mental health using assessments of depression (Patient Health Questionnaire–9 (PHQ-9); Kroenke et al., 2001) and anxiety (DSM-5 Generalised Anxiety Disorder Dimensional Scale, DSM-5 GAD-D; Knappe et al., 2013), and items focused on economic status, financial behaviours and financial wellbeing (ANZ, 2018).

#### Qualitative interview

An Autistic researcher led the design of the interview schedule (see Supplementary Table 1), which was also informed by the Phase 1 results and our review of the financial wellbeing literature outside the field of autism research. She also conducted all interviews between April and June 2021. Individual interviews took place predominantly via Zoom, although two were completed via email, according to interviewees’ communication preferences.^2^ We asked participants open-ended questions about their financial wellbeing (current and past experiences), its perceived impact on daily life, and how it could be supported. We provided participants with primary interview questions ahead of the interview to accommodate processing differences. Prompt questions were used to elicit further responses, where necessary. Spoken interviews varied in length, between 27 and 108 min (*Md* = 64.2), which were recorded with participants’ prior permission and transcribed verbatim. We returned transcripts to participants to review for accuracy and were given the opportunity to provide feedback on the results as part of the member-checking process. Participants received an AUD$50 voucher for taking part.

### Data analysis

Our analysis was informed by team members’ experience and training in psychology, education and nursing, and positionality as an Autistic advocate. We followed Braun and Clarke’s (2019) method for reflexive thematic analysis, using an inductive approach (i.e. without integrating the themes within any pre-existing coding schemes or preconceptions of the researchers) to identify patterned meanings within the data set. Our epistemological stance fits within an essentialist framework, in which our goal was to report the meanings and experienced reality of the participants. E.P. immersed herself in the data, taking reflexive notes on striking and recurring observations, reflecting on and incorporating G.H.’s reflective memos written after each interview, and applying codes to each transcript (managed in NVivo, version 12). During the familiarisation code-generation phases, it became apparent that many of the codes and potential themes were common across (low and high) groups. The same codes were therefore applied to all transcripts and the initial transcripts were re-coded, where necessary. The team liaised multiple times to review the themes and subthemes, focusing on semantic features of the data, resolving discrepancies, and deciding on the final descriptions. Analysis was therefore iterative and reflexive (Braun & Clarke, 2019).

### Community involvement

The study involved one Autistic advocate and researcher (G.H.), who had a keen interest in the financial wellbeing of her autistic peers. She was a paid member of the research team and interviewed all participants. We had anticipated that some participants’ experiences might not be so positive, so ensuring a respectful and supportive space with an autistic interviewer was critical (see Pellicano, Lawson, et al., 2022). G.H. was actively involved from the beginning of the project, resulting in collaborative decisions that improved the relevance and accessibility of materials, especially the nature and content of the interview itself, and in the analysis and interpretation of the findings. Indeed, she consistently sought to ensure that, during the analytic phase, the team adopted a strengths-based perspective and was attentive to the broader (family, societal) context influencing autistic people’s financial wellbeing.

## Results

### Quantitative

As expected, the groups differed significantly on subjective financial wellbeing, *t*(19) = 14.25, *p* < .001, Cohen’s *d* = 5.92 (Table 2). In response to the question, ‘How would you describe your current financial situation?’ on a scale ranging from ‘very bad’ (score of 1) to ‘very good’ (score of 5), almost all (*n* = 8; 88%) participants in the low group reported their current financial situation was ‘somewhat bad’ or ‘very bad’ compared to only one participant (8%) in the high group.

**Table 2.** Participants’ current financial situation for the low and high financial wellbeing groups.

	Low financial wellbeing group (*n* = 9)	High financial wellbeing group (*n* = 12)	Total (*n* = 21)
	Mean (*SD*) Range or *N* (%)		
Financial wellbeing score (out of 100)^a^	19.18 (14.81) 3.78–47.22	86.32 (6.14) 71.42–95.83	57.55 (35.60) 3.78–95.83
Current financial situation			
Very bad	4 (44%)	0	4 (19%)
Somewhat bad	4 (44%)	1 (8%)	5 (24%)
Neither bad nor good	0	1 (8%)	1 (5%)
Somewhat good	1 (11%)	4 (33%)	5 (24%)
Very good	0	6 (50%)	6 (28%)
Main source of income^b^			
Wages or salary	2 (22%)	8 (67%)	10 (48%)
Self-employed earnings	1 (11%)^c^	1 (8%)	2 (10%)
Government benefit/allowance	6 (66%)	3 (25%)	9 (43%)
Reported annual income (AUD)			
<$25,000	5 (55%)	3 (25%)	8 (38%)
$25,000–$49,000	4 (44%)	4 (33%)	8 (38%)
$50,000–$74,999	0	1 (8%)	1 (5%)
$100,000–$124,999	0	3 (25%)	3 (14%)
> $150,000	0	1 (8%)	1 (5%)
Reported savings (AUD)			
<$1000	9 (100%)	0	9 (43%)
$1000–$4999	0	1 (8%)	1 (5%)
$10,000–$19,999	0	1 (8%)	1 (5%)
$20,000–$49,999	0	5 (42%)	5 (24%)
$50,000–$99,999	0	2 (17%)	2 (10%)
$100,000–$249,999	0	2 (17%)	2 (10%)
Prefer not to say	0	1 (8%)	1 (5%)
Home loan (residence/investment)			
Yes	2 (22%)	7 (58%)	9 (43%)
No	7 (77%)	5 (42%)	12 (57%)

a Kempson et al. (2017), where higher scores indicate greater financial wellbeing; ^b^Across groups, people’s occupations varied widely, including disability support worker, administrative assistant, supermarket check-out operator, social worker, communications officer, web designer, travel agent, kitchen-hand, programme manager, secondary school teacher, social worker/counsellor, psychologist, civil servant and senior business analyst; ^c^This participant endorsed this category but also reported ‘mainly no income, very occasional self-employment at home’.

There were also clear group distinctions on employment and economic status. Almost all (*n* = 11; 92%) the high group were currently employed, compared to only three (33%) in the low group (Table 1). While most high-group participants (*n* = 9; 75%) reported wages/salary or self-reported earnings as their main source of income, only three (33%) of the low group did, with most (*n* = 6; 66%) receiving government support. It is unsurprising that all low-group participants reported receiving a total annual income before tax of less than AUD$49,000, with none having savings. Reported annual income in the high group was more varied (Table 2) – but all participants noted savings. High-group participants were also more likely to have a home loan (*n* = 7; 58%) than those in the low group (*n* = 2; 22%).

Also, while diagnoses of co-occurring conditions were common across groups, especially anxiety and depression, our low-group participants scored significantly higher than their high-group counterparts on depression, PHQ-9: *t*(19) = 3.06, *p* = .006, *d* = 1.33, and anxiety measures, DSM-5 CROSS-D: *t*(19) = 3.21, *p* = .005, *d* = 1.41, indicative of clinically-significant mental health symptomatology (Table 1).

### Qualitative

**Figure 1.**
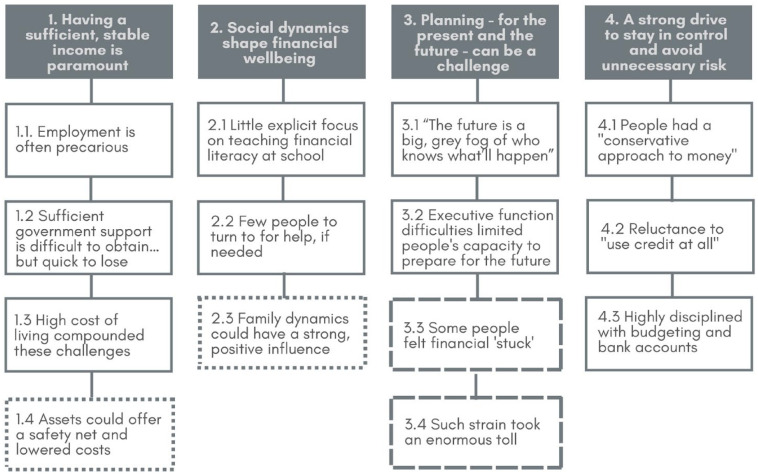
Autistic people’s experiences of financial wellbeing: themes and subthemes. Boxes with dotted line or long-dashed line indicates subthemes predominantly endorsed by those in the high or low financial wellbeing groups, respectively.

We identified four themes (Figure 1) at the community/societal (Theme 1), household (Theme 2) and individual (Themes 3 and 4) levels. Below, themes are highlighted in bold and subthemes italicised. Throughout, quotes are attributed via ID numbers (‘H’ and ‘L’ refers to high and low groups, respectively).

#### Theme 1: having a sufficient, stable income is paramount

Whether someone had access to ‘a steady stream of income’ (L978) through either employment or government support made a critical difference to their financial wellbeing. It allowed them to have enough money to cover their everyday expenses, and removed some of the uncertainty by allowing them to put some money aside, to build some financial resilience for the future.

Yet, a stable income was out of reach for many at least partly because *employment was often precarious (subtheme 1.1)*. Despite ‘wanting to be able to work’ (H272), they repeatedly reported ‘struggling to hold down a job’ (L386). The reasons for ‘not being able to keep a job for very long’ (L869) were manifold. Participants described ‘non-supportive environments’ (H272) in which they tried ‘to hide my disability’ (L869). Sometimes they felt they were ‘in the wrong kind of industries where there’s lots of high interpersonal relationships’ (H831), causing ‘a lot of stress’ (L765). Participants also found it challenging to work according to other people’s agendas. Some participants, particularly from the low group, often found themselves in casual positions ( ‘right down the bottom of the heap’; H650), which meant they did not ‘have any job security’ (L869) and no way to ‘progress’ (L978).

Mental health factors also played a part in keeping a job. Participants described ‘a lot of anxiety’ (L621) and ‘meltdowns’ (H650) at work until people either ‘got fired’ (L621) or ‘had to walk away’ (L887). This resulted in them ‘cycling through various different jobs’ (L978), which had a negative effect on mental health: ‘I’m a bit of a mess, really’ (H906). Consequently, participants described having to make choices to protect their mental health, including passing up promotions because ‘I don’t like supervising people’ (H906) or ‘working freelance’ (L793), which meant ‘not receiving a full-time wage’ (H272). One participant, after several ‘mental breakdowns’, opted to leave a ‘really high stress environment’, to work in a less-secure, less well-paid job: ‘It’s not as satisfying . . . but what I’m doing is easy’ (L869).

Government support could also be precarious. Many participants – most in the low-financial wellbeing group – described having received government support for at least part of their adult lives either related to their disability or employment status. Either way, *sufficient government support could be difficult to obtain . . . but quick to lose (subtheme 1.2)*. Many reported that accessing such benefits was ‘a stressful process’ (L978) due to difficulties completing the forms ( ‘the concepts themselves and the language are just confusing’; H316) or ‘problems with the Centrelink system’^3^ (L793).

Participants were also conscious that government supports could be reduced or even ‘taken away at any moment’ (H272). One participant described the shock when their disability support allowance was taken away: ‘It was terrifying. I had nothing, no safety net’ (H794). As a result, people preferred not to have to ‘rely on government benefits’ but emphasised ‘when you don’t have money, you don’t have options’ (H272).

The challenges of not always being able to secure a sufficient, stable income were compounded further by the *high cost of living (subtheme 1.3).* People saw ‘increased prices at the grocery checkout’ (H518) and found ‘it difficult to afford the car . . . even though we’re on disability benefits and get the regos [car registration] free’ (L621). This was especially apparent for participants living in regional and rural areas, which ‘is very expensive’ (L064). Those on government benefits emphasised that it ‘was not generally an acceptable amount’ (L386), only just enough to survive: ‘I want not to have only $10 left after everything a fortnight. And I have to afford things like medication, formula and nappy cream and stuff’ (L869). Other participants said their limited income constrained their recreational activities: ‘there’s a café that plays live music on a Friday night. I don’t even consider going, it’s too expensive’ (H272). Participants in the low group also described how ‘it becomes expensive being poor’ because ‘you can’t pay the fees for the bank account, the debit card’ (L386).

These challenges were buffered for some participants, whose *assets offer a safety net and lowered costs (subtheme 1.4)*. Many in the high group had ‘bought property’ (H906), including investment properties: ‘I turned 40 in 2018 and that year I purchased my fourth property’ (H472). Some were in a position in which they no longer ‘have a mortgage to pay’ (H794) or ‘only got a small bit on it now’ (H831). Participants explained that these assets ‘had always been our savings plan’ (H650) and made them feel financially secure: ‘I’m not going to have to worry about rent . . . it gives me some security’ (H204). Only two participants in the low group, however, reported having secured a home loan: ‘things like mortgage and insurance are not in my reality’ (L793).

#### Theme 2: social dynamics shape financial wellbeing

Participants repeatedly spoke of external factors that shaped their financial wellbeing, including family, friends and school. Most told us there was *little explicit focus on teaching financial literacy at school (subtheme 2.1)*: ‘Money is like this secret thing that no-one talks about’ (L869). One participant described ‘there was no-one teaching me how finances work, especially around the end of high school into my first job’, although she also admitted if there ‘was a financial counsellor at school, I probably wouldn’t have gone anyway; I was scared they’d laugh at me because my family was poor’ (H316). Some noted that they were introduced to bank accounts in school but it ‘was very abstract’ and there was no focus on ‘daily finances – it was always just, ‘you’ll pick it up’’ (L978). This lack of explicit teaching resulted in people feeling unprepared.

While some reported having partners who were ‘able to support me, and the kids’ (H794), most participants described having *few people to turn to for help, if needed (subtheme 2.2)*. Some reported feeling ‘genuinely scared to ask people for help’ (L978), ashamed ‘you’re not adulting properly’ (L064). This reluctance to ask for help also stemmed from ‘having been taken advantage of a bit financially’ (H650), including by friends and family. People repeatedly spoke of having ‘trouble saying “no” and standing up for myself sometimes’ (H316), ranging from ‘supporting a charity’ (L765) to ‘buying someone a car’ (L978). Some reported early, negative experiences, including ‘as a teenager, friends who’d borrow money and wouldn’t pay it back’ (H650) and ‘as a boy, I was ripped off by adults who I trusted’ (L793).

Challenging couple dynamics were also reported, with a ‘husband who was supposed to give support for the [four] children but never did’ (H794) and ‘partners who’d been leaches’ (H272), ‘bludging off me even though I was only on benefits at the same time’. (L869). Worryingly, some also recounted periods of (past or ongoing) financial abuse: ‘in my marriage, whenever I spent money, I had to explain myself’ (L064). Another described challenging family members: ‘basically, if I say, “no”, they keep begging. They actually get angry’ (L943).

Most people in the high group, however, mentioned how *family dynamics could have a strong, positive influence (subtheme 2.3)*. Sometimes family members supported them financially, ranging from ‘inheritance money’ (H518), allowing them to buy a house or a car and ‘give us a buffer’ (H650) to money for travel to ‘see family in the UK’ (H272) and other essential items, like hospital visits, ‘the tyres for the car’ (L621), or to ‘get a truck licence’ (L386). They described feeling ‘lucky’ (H272) to have this support.

Participants also described how more general, family support has often ‘been an enormous help’ (H316), ranging from filling in paperwork (L765), ‘negotiating a better price’ for utilities (H518), or someone to turn to for financial advice:

Dad told me everything about the financial stuff . . . basically, you have a system, where you have ‘daily activities’, your bills, your food shops, anything that’s high importance, as opposed to ‘splurge money’ where you just spend whatever you want. (L943)

They often reported having ‘positive examples of how to budget, how to pay bills and things like that’ (H518). Interestingly, some also recounted how having financial unhealthy role models had spurred them to ‘absolutely not want to be like my parents’ (H472):

I grew up in a family that wasn’t poor so much as just not good with money. We had several incidents of crisis, where we were panicking because a bill couldn’t be paid . . . And just remembering how hard it was to function when it felt like the foundations were always slipping and sliding, that made me really hypervigilant about my own security as an adult. (H316)

#### Theme 3: planning – for the present and the future – can be a challenge

For our participants, financial wellbeing meant having ‘a proper plan for the future’ (L943), giving people ‘a sense of confidence’ (H272). While they were aware that goals and ‘directions change as you move through life’ (H831), many were concerned that ‘*the future is a big, grey fog of who knows what’ll happen*’ *(subtheme 3.1)*. This concern was particularly apparent from older participants: ‘anybody at this stage of life, from their 50s on, is going to start thinking about mortality – about how more limited your options are’ (L793). They were worried about their partners, ‘who’s not always physically well’ (H204), and of ‘remaining independent and looking after myself’ (H316). Some, particularly those in the high group, felt secure and were ‘looking forward to being pension age, because I know it’s going to be quite comfortable for us’ (H650). Others, however, were far less certain: ‘How do we plan for our retirement? How do I understand the super[annuation] stuff because I don’t understand it and I don’t know how to make decisions about what we should do with it’ (H204). They also spoke of needing to adjust their standard of living as retirement loomed: ‘because the pension isn’t very much, that will certainly change things for us . . . I will need to work as long as possible’ (H794). Those who still had ‘a long way to go’ until they retire had ‘no concept of how much money I should have, and no concept of how to figure that out either’ (L978).

Those with children – especially those who are Autistic – were also worried about what ‘her future might be and is there a way to progress her into some activity that might give her some kind of independence or financial stability’ (H272). They felt ‘a bit of protectiveness in terms of trying to set him up financially’ (H204). One participant highlighted intergenerational issues:

‘They [my children] have an understanding of money, but they’re Autistic, and there are certain barriers that come with that that make it difficult . . . so it really does impact on your wellbeing, not just for us, but family, their family’ (H794)

A minority of participants, all in the high group, felt ‘very capable of planning for the future, constantly forward thinking’ (H042), even ‘hyperplanning’ (H650). But, for most, being able ‘to envisage financial security and being able to appropriately plan for this’ (L887) often felt out of reach: ‘sometimes tomorrow is a pie-in-the-sky concept’ (H316). This was especially for our low-group participants, who did not always have enough money to meet immediate basic needs: ‘I don’t even think about living life large or building a nest egg for comfortable retirement. I’m purely focused on ensuring safety and continued existence’ (H316). They felt their difficulties with ‘organisation, time management, narrow focus, planning, emotion regulation’ (L887) or *executive function limited people’s capacity to prepare for the future (subtheme 3.2)*: ‘I can’t do the executive function stuff’ (H794).

Participants emphasised how anxiety could often exacerbate challenges with financial planning. Maintaining a routine often helped with financial planning, but when that ‘routine starts getting thrown out, all my functions seem to off, and that, through the anxiety, makes a little cascade of poor decisions and poor financial choices’ (L064). The cognitive effort ‘and constant alertness required [to plan] could be exhausting’ (H272), and the sense of overwhelm with ‘so many roadblocks’ (L064) could also have negative effects, including not ‘remembering to pay bills or call Centrelink’ (L869). It could also be debilitating, leading to inertia ( ‘I keep needing to do things, but I can’t do anything’; L869) and ‘slipping into burnout’ (L887).

Given that participants in the low group simply did not have sufficient income to meet their and/or their children’s basic needs, *some felt financially ‘stuck’ (subtheme 3.3)*: ‘I would like to thrive, but I get so stuck on the “hey, just survive”, that the thriving seems like it’s reaching too far at the moment’ (L978). Participants described having previously lived or currently living in poverty – ‘a vicious cycle that it’s so impossible to break’ (L386). One participant remembered how ‘it was a constant battle to have enough money to pay for things. If you’ve got to think all the time, how am I going to pay for that? How do I cut back here so I can cover this? It takes up a lot of energy and a lot of life’ (H794).

Participants recounted how *such strain took an enormous toll (subtheme 3.4)*. One described how ‘it’s very hard to have the time and the money and the energy and the executive function to cook a meal . . . she [baby] doesn’t have a balanced diet – and neither do I’ (L869). Some repeatedly spoke of how ‘it actually impacts my mental health . . . I get the money but then it’s instantly gone’ (L943). This sense of feeling financially vulnerable had people ‘locked up tight, stressed, focused on just how to get by rather than wellbeing’ (H272). They described relentlessly ‘having your optimism and your enthusiasm kicked out of you’ (L793). Some reported trying to access ‘outlets to get out of that headspace’ (L765), but many felt like they were on a ‘rollercoaster of not coping’ (L064). They reported ‘thinking that you should be able to do better, you should be able to provide for yourself’ (H794) and those feelings of internalised shame were often too much to bear: ‘I just feel like everything that I am is wasted’ (H272). They felt ‘worthless’ (L887), ‘pretty powerless’ (L793) and invisible: ‘you’re just existing and people can’t actually see’ (L064). One participant (L978) explained that this was further compounded by having to live in a world that is not set up for autistic people: ‘I’m expending a lot of that energy just to survive in the outside world as well. It feels like a double whammy’.

#### Theme 4: a strong drive to stay in control and avoid unnecessary risk

Despite all these challenges, participants described attitudes and behaviours that suggested they were regulating their financial behaviour, even if they did not *feel* they could manage. While some described instances of ‘impulse buying’ (L887), overall, *people had ‘*a conservative approach to money’ *(subtheme 4.1)*: ‘even though I have more money now, I’m still Scroogey about it’ (H042). They reported being ‘more of a saver than a spender’ (H518), which meant some people didn’t spend much ( ‘I deny myself stuff’; H650) because they ‘hated that feeling of not having money’ (H272). Many had had ‘experiences of having nothing, so, yes, it just makes me feel safer if I’ve got it in the bank’ (L978), ‘in case disaster happens’ (H204). Some acknowledged, however – if only fleetingly – that they ‘wish[ed] they had the courage to be a bit more flexible’ (H906) with their spending.

This preference to save, not spend also carried over to people’s *reluctance to ‘use credit at all’ (subtheme 4.2)*. Our participants preferred ‘not to run the risk’ (L978) because ‘people can get into trouble with that’ (H518). Indeed, participants were ‘terrified of debt and a credit card that you can’t pay off at the end of the month’ (H906). And if they did have a credit card, they ‘rarely used it’ (L064) or would only use it ‘for travelling’ (H472) or ‘because I wanted to buy stuff online’ (H316). In those cases, they would ‘pay my credit card off in advance’ (H272); ‘I’ve never got anything outstanding’ (H831). Some people reported wanting a credit card but had ‘never been approved . . . when you’re on JobSeeker [government support], the banks won’t allow you to touch that, it’s too low’ (L386). One participant reported using a hire purchase scheme ‘as an emergency thing’ (L943).

Participants were also *highly disciplined with budgeting and bank accounts (subtheme 4.3)*. They reported a tendency ‘to watch my bank accounts’ (H272), with some ‘checking the bank balances every day’ (H042). They often described ways of managing their bank accounts to pay bills and to save a bit if they could: ‘I have a bank account that’s like a saver account. So, I always have an amount, just $50, that goes in every month’ (H272). They were also intentional about budgeting ( ‘I write a list and budget and stuff’; L869), sometimes overcoming deep-rooted lack of confidence with maths ( ‘I’m not very good with figures’; H794) to manage money effectively. Some people’s challenges in this area nevertheless persisted: ‘As soon as there’s numbers and money involved, all my cognitive capacity just fucks right off’ (H316). As such, they emphasised their desire to ‘feel more in control’ (H316) and develop ‘skills with budgeting and planning’ (L887).

## Discussion

In this study, we sought to understand the subjective experiences of financial wellbeing and the factors that influenced them from the perspective of autistic adults who could sustain the demands of an in-depth qualitative interview. Overall, our interviewees felt that financial wellbeing meant having enough money to make ends meet, to save for unexpected future expenses and not to have to worry about financial matters. These characteristics map closely onto conceptualisations of financial wellbeing in the general population (Kempson et al., 2017; Salignac et al., 2020). Many autistic participants, however, felt they were often beyond reach. Precarious employment and government support meant they did not have a sufficient stable income for day-to-day expenses and a safety net for the unexpected. Consequently, they were limited in their choices. These difficulties came despite often being extremely disciplined in their budgeting strategies. Ultimately, the stress of financial insecurity was often too much to bear, especially when they were unable to rely on family members and friends for direct or indirect financial support.

Our interviewees emphasised how much having a stable, secure income mattered to them, similar to non-autistic people (ANZ, 2018; Comerton-Forde et al., 2018; Kempson et al., 2017; Muir et al., 2017). Unfortunately, despite wanting an opportunity to work and possessing key skills prized by employers, including loyalty, reliability and attention to detail (Austin & Pisano, 2017; de Schipper et al., 2016; Russell et al., 2020; Scott et al., 2017), autistic people face substantial challenges in gaining and sustaining employment, even relative to other disabled people (Office for National Statistics, 2021; Roux et al., 2015; Scott et al., 2019). Among the autistic adults who eventually obtain employment, they are all-too-often in positions that fail to match up with their skill set and abilities (malemployment) or for which they are overqualified (underemployment), two scenarios to which our participants clearly attested. They also highlighted their work-related challenges, especially regarding maintaining employment, were not always related to their own, individual difficulties, but were due to inhospitable work environments. Again, this finding is consistent with studies documenting the challenges autistic people face in the workplace, including negative experiences with, and sometimes bullying by, colleagues (Baldwin et al., 2014; Hendricks, 2010), failure to have their needs met through reasonable adjustments (Petty et al., 2023), and experiences of stigmatisation and discrimination (Romualdez et al., 2021; Thompson-Hodgetts et al., 2020). These challenges often took their toll, which meant that either their career progression stagnated, they were relegated to less-secure, causal positions, or were driven to unemployment. While there are increasing efforts around the world to enhance the inclusivity of workplaces and work practices (e.g. Flower et al., 2019; Remington et al., 2021), it was clear from our respondents that these initiatives need to be extended.

Employment will not be a route to financial wellbeing for all autistic people, however, just as it is not for non-autistic people (see K. A. Anderson et al., 2020). Our participants emphasised how they wish government supports to be more generous, accessible, and more predictable. Unfortunately, and consistent with a number of advanced democracies, the Australian disability support pension (AUD$25,155 p.a.; Services Australia, 2022b) and unemployment benefit (AUD$16,367 p.a.; Services Australia, 2022a) at the time of writing sit on or substantially under the Australian poverty line (AUD$23,764 p.a.; Davidson et al., 2020), respectively, and fall well below average full-time earnings (AUD$90,917 p.a.; Australian Bureau of Statistics, 2022). The hardship does not stop there, however. Participants repeatedly described the processes of applying for and accessing these funds were complex, confusing and burdensome. There has been much written on the overly bureaucratic nature of benefits systems and the stigma it can cause disabled people (e.g. Saffer et al., 2018). Experiencing stress – and financial scarcity in particular – together with additional physical and mental health problems are thought to compound further people’s challenging experiences of interacting with government services and systems. It is unsurprising, therefore, that our autistic participants found such interactions so onerous, given that many reported experiencing executive problems, co-occurring physical and mental health issues and financial insecurity. This ultimately means that autistic people are more likely to need government assistance, but they are less likely to have the resources to navigate the (executively-demanding) systems necessary to secure such assistance (Christensen et al., 2020). Such issues will affect autistic people with *and* without a formal diagnosis, although those without a diagnosis – and therefore ineligible for some government supports (such as insurance benefits) – may be disadvantaged further still.

Family members sometimes helped our participants to manage all the ‘red tape’ associated with navigating government services and systems. In fact, family members, along with partners and friends, were felt to be a strong influencer of an individual’s sense of financial wellbeing, consistent with an ecological systems approach (see Salignac et al., 2020). On one hand, our mostly high-group participants emphasised how support from family members made a positive difference – either through financial assistance or financial guidance.

On the other hand, we also heard, largely from those in the low group, about the detrimental effects of a lack of social support and how family, partners and friends could have a negative influence on participants’ financial wellbeing, through being taken advantage of or even economic abuse. This combination of an absence of positive social support *and* the presence of negative social influences meant that they had few external resources to rely upon to buffer financial insecurity and, ultimately, their wellbeing. Participants in the low group had significantly greater levels of anxiety and depression than their high-group counterparts, and they spoke at length about how feeling financially vulnerable had damaging effects on their mental health. Recent findings in the general population indicate that people with lower financial wellbeing report poorer mental and physical health (ANZ, 2021). This may be driven by the well-established link between health and income: those on higher incomes tend to be exposed to fewer risk factors associated with poor health, including access to better housing, neighbourhoods, nutrition and exercise, and are less likely to experience stress and social isolation (e.g. Benzeval et al., 2001; Clougherty et al., 2010). Poor health can also affect a person’s ability to work and their level of earnings, but longitudinal studies suggest that the impact of income on health is far greater than health on income (e.g. Beckett et al., 2000). While level of income matters, how one feels about their financial situation (i.e. their financial wellbeing) is also critical: those with low incomes *and* who report challenges managing with these incomes were more likely to report poor health (Arber et al., 2014). Consistent with these general population findings, our Phase 1 analyses demonstrated that autistic adults with greater depressive symptoms also reported lower subjective financial wellbeing (Cai, Hall, & Pellicano, 2023). More research is needed to understand the causal nature of the link between subjective financial hardship and poor mental health in autistic people, especially during periods of economic uncertainty (Andersen & Reeves, 2022).

Despite all these challenges, our autistic interviewees – across both high and low financial wellbeing groups – tended to report prudent financial attitudes and behaviours. They were risk averse, avoiding access to credit cards or payday loans; highly diligent with their budgeting; and actively saved money when possible. Such behaviours are often thought to make an important contribution to financial wellbeing both in the general population (Comerton-Forde et al., 2022) and in autistic people (Cai, Gallagher, et al., 2023). It is perhaps unsurprising that our autistic participants show an advantage in this regard. Research has shown that autistic people make more logically-consistent, rational decisions (de Martino et al., 2008; Goslin & Moutier, 2018), are more circumspect in their decision-making, sampling more information prior to making a decision (Brosnan et al., 2014; Vella et al., 2018) and more deliberate in their reasoning (Brosnan et al., 2016; Farmer et al., 2017; Shah et al., 2016). This distinctive way of approaching decisions, and financial decisions in particular, may serve to buffer some negative effects of financial insecurity.

Notwithstanding these strengths, our autistic participants – particularly those in the low group – wanted to enhance their financial knowledge and skills to feel more in control. Unfortunately, many reported they were not taught about money matters in school, and had little opportunity to access formal advice, support and education on financial management, especially early in adult life. Autistic young people have also reported feeling ill-equipped to achieve their financial goals and wanted more education and resources on managing their finances (Cheak-Zamora et al., 2017). Providing financial education at school is especially important for those who may not be exposed to it at home. While there are increasing efforts to promote the financial literacy of non-autistic people, especially in workplaces (Lusardi, 2019), such efforts need to be inclusive of autistic people. Being outside of higher/further education and employment means that many autistic people might also miss out on the informal and formal teaching in financial management available to other adults. There have been some efforts to provide freely-available and easy-to-access financial education for autistic adults. The UK’s National Autistic Society offers a ‘Managing Money’ module and Autism Speaks has developed a Financial Planning Tool Kit. Future research should examine the effectiveness of these modules/toolkits with autistic people across a range of age groups.

The banking industry also has a key role to play in enhancing financial inclusion (Cai & Gibbs, 2020; Livingstone, 2007), including accessible branches (Alix, 2017; Cai & Gibbs, 2020), financial advice, financial products and adult financial education programmes. One study examining autistic people’s views on ANZ’s MoneyMinded financial education programme found that while they were interested in learning about income and tax, how to make financial decisions and understanding debt, they were also clear about *how* such learning might be most effective, including having step-by-step instructions, examples that illustrated autistic people’s experiences specifically, and having someone available for support and advice (Russell et al., 2017). More research is needed to understand how financial education programmes (in schools, communities and organisations) can best assist autistic people – and their carers – to achieve financial wellbeing and financial inclusion.

### Limitations

Our research is not without limitations. First, it is possible that our sample was biased in several ways. The preponderance of women in our sample is not consistent with current epidemiological data (Loomes et al., 2017), but may be related to the higher participation of women in online autism research (Arnold et al., 2019; Kapp et al., 2013). Our participants were also predominantly of white ethnic background and well-educated. Research outside the field of autism has shown that people from ethnic minorities, who in many circumstances experience low income, discrimination, social exclusion and poor mental health, also suffer from high financial stress (Evans et al., 2023). It is therefore likely that the combination of being autistic and being marginalised in other ways (including disability, race, ethnicity, gender and/or sexuality) could present compounding barriers to good financial wellbeing. Future work should focus on how intersectionality and social influences affect autistic people’s financial status and financial wellbeing.

Second, since only one of our participants reported an intellectual disability, it is likely that our recruitment methods led to a bias towards those who were willing and able to convey their experiences through in-depth interviews, especially those who used traditional forms of communication and were not accessing more specialised supports. One critical next step for future research is to examine the financial wellbeing of those autistic people who are seldom heard in autism research, including purposively sampling autistic people with intellectual disability, those who use non-traditional forms of communication, those from minority ethnic backgrounds and/or those who require high levels of care and support. Doing so will require researchers to go beyond standard semi-structured interviews, creating innovative methods in order to respond flexibly to their individual needs, preferences and interests (see Nind, 2008; Overmars-Marx et al., 2018).

Third, it is noteworthy that most of our sample received their autism diagnoses in adulthood. There is emerging evidence that late-diagnosed autistic adults differ from their childhood-diagnosed counterparts, with late-diagnosed adults reporting higher autistic traits and poorer quality of life (Atherton et al., 2022), and more co-occurring psychiatric diagnoses (Jadav & Bal, 2022) than those diagnosed as autistic earlier in life. It will therefore be important to determine whether the pattern of findings reported here also reflects the experiences of autistic people diagnosed in childhood.

Finally, while we sampled people across a range of ages, most participants were in mid-adulthood, which meant we were unable to consider the temporal nature of autistic people’s financial wellbeing – the ways in which a person’s financial wellbeing can change over time and at different life stages (e.g. transition to adulthood; transition to retirement) and how such changes are influenced by individual and social factors (cf. Salignac et al., 2020).

### Conclusion

This study revealed how much money matters in autistic people’s lives – and how financial wellbeing is influenced by a range of individual and broader social (household, community and societal) factors. Future research should investigate further ways for autistic people to secure more reliable incomes, and how they can be supported as they seek to maintain financial sustainability in often demanding and unpredictable personal and societal circumstances.

## Supplemental Material

sj-docx-1-aut-10.1177_13623613231191594 – Supplemental material for Autistic adults’ experiences of financial wellbeing: Part II Supplemental material, sj-docx-1-aut-10.1177_13623613231191594 for Autistic adults’ experiences of financial wellbeing: Part II by Elizabeth Pellicano, Gabrielle Hall and Ru Ying Cai in Autism

## Notes

1. The reasonably low uptake rate from those with particularly low subjective financial wellbeing scores meant that we extended our invitations to those who obtained scores between 0 and 50.

2. Face-to-face interviews were not possible due to prevailing COVID-19 stay-at-home orders during the period of this study. Our email interviews involved a series of asynchronous, researcher probe-participant response interchanges in which the researcher began by emailing the primary research questions to the participant in a Word document and allowing the participant to respond. Once the participant responded, they were asked additional follow-up questions and prompt questions, as per our interview schedule. These methods followed Dowling (2012) and Hershberger and Kavanaugh’s (2017) guidance as well as our own experience of conducting successful email interviews in previous studies (Pellicano, Brett, et al., 2022).

3. Centrelink is the Australian federal agency that distributes welfare funds.
