# Supplementary material for: Autistic adults’ experiences of financial wellbeing: Part II
Source: Autism. 2023 Oct 5;28(5):1090–106. doi: 10.1177/13623613231191594 (PMC11067415; doi:10.1177/13623613231191594)
Supplement: sj-docx-1-aut-10.1177_13623613231191594 – Supplemental material for Autistic adults’ experiences of financial wellbeing: Part II [file sj-docx-1-aut-10.1177_13623613231191594.docx]

**Supplementary Table 1.** Semi-structured interview schedule. Participants were asked all primary questions, which they received prior to the interview. Prompt questions helped the interviewer to gain more detailed information, if necessary.

| **Primary questions** | **Question Prompts** |
| --- | --- |
| 1. Tell me a bit about yourself. | When did you get an autism diagnosis? Self or formally diagnosed?  What about your family? Do you have a partner or children, that do or don’t live with you?  How do you spend your days? For example, are you working, studying, volunteering or receiving government benefits?  Who do you interact with, and what do you do in a regular day? |
| 2. Now moving on to financial wellbeing. What does the term ‘financial wellbeing’ mean to you? Why do you think that? | How would you describe your current financial wellbeing? How do you feel about that?  During your life, how have you leant about finances?  Do you normally spend, save or a bit of both? Strategies? What has influenced this?  How has study or work been for you?  Who do you turn to for advice on finances?  How confident do you feel to manage your current or future financial wellbeing? |
| Check if participant needs a break | |
| 3. What might life look or feel like, when you have good financial wellbeing? | Do you think it is the same for non-autistic people?  Are there things about being autistic that make it difficult or easy to achieve good financial wellbeing? If yes, what are they?  Are there other aspects of your life (other than being autistic), that can make it difficult or easy to achieve good financial wellbeing? (e.g., having attention problems, mental or physical health issues, a family to take care of) |
| 4. How do you feel about your life in general? What’s tricky or easy? Do your finances impact how you feel, and what you do? If so, how? | [probe for positive or negative impacts]  Does your financial wellbeing impact your:  a. physical health  b. mental health  c. the amount/type of support you receive  d. access to health, education  e. family situation  f. leisure and passions? |
| Check if participant needs a break | |
| 5. Would you like your financial wellbeing to be different? What could support this? | Do you receive some support from anyone? If so, what could the people who support you, do to make it better?  What things could organisations (like banks or government) do, to help you improve your financial wellbeing?  How would you like to learn about these things? (e.g., through the internet, through face-to-face meetings etc.). Are there organisations you would trust to provide this information? |
